# Algorithmic Political Bias in Artificial Intelligence Systems

**DOI:** 10.1007/s13347-022-00512-8

**Published:** 2022-03-30

**Authors:** Uwe Peters

**Affiliations:** 1 Center for Science and Thought, University of Bonn, Bonn, Germany; 2 Leverhulme Centre for the Future of Intelligence, University of Cambridge, Cambridge, UK; 3 Department of Psychology, King’s College London, London, UK

**Keywords:** Algorithmic bias, Artificial intelligence, Political bias, Political psychology

## Abstract

Some artificial intelligence (AI) systems can display algorithmic bias, i.e. they may produce outputs that unfairly discriminate against people based on their social identity. Much research on this topic focuses on algorithmic bias that disadvantages people based on their gender or racial identity. The related ethical problems are significant and well known. Algorithmic bias against other aspects of people’s social identity, for instance, their political orientation, remains largely unexplored. This paper argues that algorithmic bias against people’s political orientation can arise in some of the same ways in which algorithmic gender and racial biases emerge. However, it differs importantly from them because there are (in a democratic society) strong social norms against gender and racial biases. This does not hold to the same extent for political biases. Political biases can thus more powerfully influence people, which increases the chances that these biases become embedded in algorithms and makes algorithmic political biases harder to detect and eradicate than gender and racial biases even though they all can produce similar harm. Since some algorithms can now also easily identify people’s political orientations against their will, these problems are exacerbated. Algorithmic political bias thus raises substantial and distinctive risks that the AI community should be aware of and examine.

## Introduction

AI systems, which are computer programs that can find patterns in vast amounts of data and may automatically improve their own performance through feedback, are increasingly making important judgements and decisions (Lee et al., 2019). They are now being used to decide, for instance, whether a job applicant is suitable for a vacancy (Bogen, 2019), whether someone is eligible to receive a loan (Khandani et al., 2010), how long a convict should stay incarcerated (Berk et al., 2016), or what medical diagnosis a patient should receive (Jiang et al., 2017).

Yet, while AI systems enjoy an aura of objectivity and accuracy (Kahneman et al., 2016), they can show *algorithmic bias*, i.e. a tendency to not merely neutrally transform or extract information from data but to operate on it in ways that deviate from a normative (moral, statistical, social, etc.) standard such that one kind of individual or group is unfairly privileged over another based on aspects of their social identity (Danks & London, 2017). For example, at Amazon, a now scrapped AI algorithm for job recruitment systematically downgraded women’s CVs and so displayed a gender bias (Vincent, 2018). Elsewhere, an algorithm that predicted whether defendants would re-offend gave higher risk scores to African-Americans than to Whites although both groups were equally likely to re-offend (Rudin et al., 2020). Relatedly, some algorithms that powered facial recognition AI systematically misclassified darker-skinned complexions (Buolamwini & Gebru, 2018), or mislabelled Black men as ‘primates’ (Mac, 2021).

There has been a flurry of research on algorithmic bias (Fazelpour & Danks, 2021). Much of it focuses on algorithmic gender and racial biases (Noble, 2019; West et al., 2019). No doubt, they are important to understand and eradicate. But algorithmic biases against other dimensions of social identity (the features in virtue of which one belongs to a certain social group) remain largely unexplored. This may be problematic. There might be significant differences between them that go unnoticed but are critical for evaluating the potential risks of algorithmic bias. For example, algorithmic bias against some dimensions of social identity might be much harder to recognize and counteract than algorithmic bias against others.

The goal here is to make progress with respect to this issue by focusing on an algorithmic bias that targets a dimension of social identity other than gender and racial group membership, namely people’s *political orientation*, i.e. their identity as liberal, conservative, moderate, Marxist, anarchist, and so on (Jost et al., 2009). Considering political orientation in this context is especially interesting because whether individuals we meet are ‘engines of change or preservers of the status quo’, and so whether they are politically liberal vs. conservative, is one of the most frequently invoked and ‘fundamental dimensions on which [we] spontaneously distinguish social groups’ (Koch et al., 2016, p. 702). Unsurprisingly, there has been much research in psychology on people’s political biases, specifically on their explicit or implicit stereotypes and negative feelings towards individuals or groups based on their political orientation (Finkel et al., 2020; Iyengar et al., 2019). However, *algorithmic* political bias, an algorithmic bias in AI systems that targets the political orientation of individuals, groups, or contents (e.g. website content, claims, arguments), has not been investigated in this context or in work on AI yet.

This paper aims to do so. It will argue that algorithmic political bias (e.g. in job recruitment situations) can arise in some of the same ways in which algorithmic gender and racial biases emerge but it differs from them in ways that create epistemic and ethical challenges unappreciated the field of AI so far. The reason is that there are (within a democratic society) strong social norms that constrain gender and racial biases across domains but there are no equally^1^ powerful and wide reaching norms against political biases. Political biases can thus more forcefully influence individuals. This increases the probability that these biases become transferred to algorithms and makes it harder for people to detect and eradicate algorithmic political biases than algorithmic gender and racial biases even though they all can create similar harm. Furthermore, some algorithms can now readily determine people’s political orientations against their will. This further amplifies the problems that algorithmic political biases pose.

However, the argument that I will develop for these claims is compatible with the view that algorithmic gender or racial biases operate more frequently, are overall more harmful, and should more urgently be tackled than algorithmic political bias. The point here is that algorithmic political bias, too, creates significant problems while posing special challenges that we risk overlooking if we treat algorithmic bias as a homogeneous phenomenon that has the same functional profile when it is targeting different aspects of social identity.

It may be suggested that the intended comparison between biases against gender, racial identity, and political orientation is itself problematic because gender and racial identity are not a matter of choice whereas political orientation is. However, there is evidence that political orientation is not always fully chosen but partly biologically determined (Funk et al., 2013; Kalmoe & Johnson, 2021; Tilley, 2021). Additionally, in some cases, racial identity and gender, too, may be a matter of choice (Desmond-Harris, 2014; Whittle & Milbank, 2017). It is thus worth remaining open-minded with respect to the nature of these three dimensions of social identity.

Section 2 clarifies key concepts and the kind of algorithmic political bias that will be relevant for the argument to follow. Section 3 considers how AI systems might acquire this bias. Section 4 highlights differences between human gender and racial biases, on the one hand, and political biases, on the other, before relating these points to algorithmic political bias. Section 5 briefly discusses distinctive challenges for mitigating this bias.

## Conceptual Clarifications

### Political Bias

In human cognition, political bias is not a singular, unified psychological phenomenon but might be a conscious (explicit) or unconscious (implicit) thought or affective process targeting different political orientations (Iyengar et al., 2019; Jost et al., 2009). The focus here will be on political bias that targets two central and internationally common political positions, namely the liberal or politically left-wing viewpoint, and the conservative or politically right-wing orientation (Heywood, 2015).

While the particular features of these two positions may differ between countries, studies suggest that all ‘around the world [there is a] recurrent association between the left, egalitarianism’, progress, personal/social freedom, internationalism, and state intervention to regulate the economy, whereas the ‘right is invariably identified’ with traditional values, authority, order, nationalism, ‘market liberalization, and lesser state intervention’ in the economy (Rosas & Ferreira, 2013, p. 9; Caprara & Vecchione, 2018). People on the left and right are known to battle each other over political power in public domains (Bobbio, 2016). But the two camps are not homogenous and always clearly demarcated. Some positions on the left and right might overlap (Crawford et al., 2017), and both sit on a spectrum containing many different positions ranging from slightly left- or right-leaning to extremely left- or right-leaning viewpoints (Heywood, 2015).

The debate on political bias against people on the left or right remains a sensitive topic (Hershey, 2020). The reader might share one of the two orientations and wonder which side this paper will take. I will not support either side here but aim to highlight a general problem: People on the left and right have equal reasons to be concerned about algorithmic political bias and their ability to detect and eradicate it.

### Algorithmic Political Bias and Related Phenomena

Algorithmic political bias occurs when an AI system’s output tends to violate a normative (moral or social) standard resulting in one kind of individual, group, or content being unfairly privileged or discriminated against based on their political orientation. The meaning of ‘unfair’ and ‘fair’ in the context of algorithms is commonly ‘judged against a set of legal or ethical principles, which tends to vary depending on the local government and culture’ (Fletcher et al., 2021, p. 7). There is currently ‘no clear agreement [in the AI community] on which definition [of ‘fairness’] to apply in each situation’ (Verma & Rubin, 2018, p. 1). I will thus work with the rough dictionary notion of fairness as ‘impartial and just treatment’ (Fletcher et al., 2021, p. 7), and illustrate what is meant by ‘algorithmic political bias’ here by distinguishing the target phenomenon from two related ones that will not be discussed in this paper.

First, many websites employ personalization algorithms to provide website users with content similar to what they previously viewed so as to keep them engaged (Kozyreva et al., 2021). Markers of political orientation might become predictors for relevant information for a given individual (Robertson et al., 2018) and be used by personalization algorithms to selectively present some contents (or people and groups) to that individual and ignore others because of their political orientation (Le et al., 2019; Thorson et al., 2021). If impartial processing is viewed as a normative standard, then these algorithms deviate from it, and their content filtering might be interpreted as a politically biased computation. This is debatable, however. I will thus set this phenomenon aside here.

Second, while personalized content filtering might be acceptable, the algorithms behind it may also realize certain political goals by website operators (e.g. Facebook; Manhoo, 2016). Some algorithms could be specifically designed to correct for apparent bias and discrimination to reduce social injustice (Tene & Polonetsky, 2018). Similarly, social media algorithms may be trained to proactively block or remove some political, dangerous, or untrustworthy information (e.g. Nazi content) (Cobbe, 2020). This need not be problematic. But policy-directed filtering algorithms can also violate legitimate user expectations of policy-neutrality and unfairly discriminate against some individuals (Olla, 2021), groups (Reeds, 2020), or contents based on their political orientation (Tene & Polonetsky, 2018). This may be interpreted as algorithmic political bias. Isolating the relevant cases is challenging, however. This phenomenon, too, will thus be set aside here.

Instead, the focus will be on the following two kinds of processes. Algorithms are not perfect but may commit errors when classifying people. Their accuracy is often related to the size of the datasets that they are trained on: a smaller training dataset commonly produces more inaccuracy, making groups underrepresented in datasets more vulnerable to classification errors (Mohri et al., 2018). Consider, then, the finding that in US academia, overall, ‘Marxists are rare’ (< 18% of all professors; Gross & Simmons, 2014, p. 33). A hypothetical AI algorithm classifying people as US academics or non-academics (e.g. for hiring purposes) and able to detect people’s political orientations in its training data (e.g. via CV cues; see Section 3) and use them as predictors will have a smaller dataset linking Marxists with the label ‘academic’, increasing the chances that it subsequently misclassifies them as non-academics. The classification-error^2^ metrics for different (Marxist vs. non-Marxist) individuals would indicate an algorithmic political bias.

Similarly, consider a conservative applying for an AI CEO vacancy at a company in Silicon Valley. Since conservatives tend to be underrepresented in this work environment (Seetharaman et al., 2017), being conservative can become a relevant predictor that a job-recruitment algorithm may pick up from its training data (e.g. via political affiliation cues on CVs), treating it as a proxy for hiring outcome embedded implicitly in previous human decision-making (see Section 3). For the algorithm, this political orientation will have a negative statistical effect on the predicted probability to hold an AI CEO position. If the algorithm acquired and used that information in its recruitment decisions such that otherwise equally qualified candidates get a worse treatment in hiring, it would display political bias.

The focus here will be on these two kinds of cases, i.e. cases in which algorithms make socially relevant predictions or decisions that are based on political orientation in contexts where this feature should be irrelevant. Unless otherwise indicated, the term ‘algorithmic political bias’ will henceforth designate these types of computational processes. Are such biases *real*? The following arguments suggest that they can easily emerge in many AI systems.

## How Might Algorithmic Political Bias Arise?

The here relevant AI algorithms are mining data according to models that they have formed through machine learning (ML) (Burrell, 2016). To illustrate a case of supervised ML^3^ with a toy example, consider the training of an algorithm for hiring decisions. The algorithm is fed with CVs of past applicants as training data, where these CVs were classified by human agents with labels such as ‘suitable candidate’ or ‘unsuitable candidate’. The algorithm is then instructed to extract ‘suitable candidate’ features from the CVs and develop a predictive model for identifying such candidates in the training data and, subsequently, in new applicants’ CVs, which it did not previously encounter (Li et al., 2020).

Importantly, while CVs often explicitly state, for example, job qualification, work experience, gender, and racial identity, they might also contain direct or indirect cues of *unstated* features (Lee et al., 2019), which may include political orientation. For instance, political campaigning experience (e.g. for a socialist cause), publications (defending particular political views), previous jobs (e.g. liberals being academics, conservatives being entrepreneurs; Swanson, 2015), university degrees (e.g. liberals studying philosophy, conservatives economics; Gross & Simmons, 2014), zip codes (e.g. liberals living in cities vs. conservatives living in rural areas; Parker et al., 2018), or links to personal websites, or social media (LinkedIn, Instagram, Facebook) may provide human recruiters with information about and proxies for applicants’ political orientations (Roth et al., 2020). Social media contents, in particular, make it easy to discern people’s political orientation via, for instance, implicit cues (‘Black Lives Matter’ hash tags, ‘Choose Life’ signs, etc.; ibid). And just as human recruiters (about 43% of US job recruiters use social media to evaluate applicants; Henderson, 2018), recruitment algorithms, too, might be trained to process these data alongside people’s CVs.

Crucially, both explicitly stated and proxy features may correlate with ‘suitable (or unsuitable) candidate’ status. And so, while algorithms will during their training recognize relationships between applicants’ *qualifications* and successful outcomes, they can also detect correlations between qualification-independent factors—e.g. gender, racial identity, or proxies of political orientation—and outcomes (Barocas & Selbst, 2016). They may then form models that take these factors as predictors and subsequently treat different groups unequally based on gender, race, or political orientation rather than relevant qualification differences (Köchling & Wehner, 2020). Moreover, since socially sensitive variables such as, for instance, racial identity or political orientation might correlate with innocuous ones (e.g. zip codes), even when the algorithms are prevented from using sensitive variables as predictors, they could still latch onto these unproblematic factors. By treating them as proxies for the sensitive ones, they can produce the same outputs as before (see also the ‘proxy problem’ in Johnson, 2021).

But how might systematic correlations between irrelevant (incl. proxy) features and negative outcomes arise in the first place and become picked up by algorithms? There are at least three ways in which this could happen (Danks & London, 2017).

1. Mislabelling of the AI training data

In previous recruitments, due to implicit bias, employers might have consistently (and inadvertently) in their CV labelling downgraded candidates with degrees from women’s, historically Black, or conservative/liberal institutions, certain political volunteering work, previous jobs, or certain political social media cues. Job-recruitment algorithms trained on data from these past decisions, too, may then take these features as predictors of low hiring success and disfavour certain gender, racial, or political groups with credentials otherwise equal to other applicants even if no employer ever explicitly indicated the problematic predictor-outcome relationships in previous decisions (Barocas & Selbst, 2016). That is, just as implicit gender and racial biases might lead people to mislabel AI training data (Lee et al., 2019), political biases could do so too. Indeed, using the same methodology commonly employed to identify implicit gender and racial biases, i.e. the Implicit Association Test (IAT) (for details, see Kurdi & Banaji, 2021), Iyengar and Westwood (2015) found that Democrats and Republicans showed strong automatic associations between negative words and Republican and Democrat contents (e.g. party symbols), respectively. Moreover, when Democratic and Republican participants were asked to decide on the award of a scholarship to one of two students based on their CVs (containing either a Democrat or Republican identity cue), most participants selected the student with their own political orientation even when the student with the opposite orientation had higher grades (for more evidence of political bias in hiring contexts, see Gift and Gift (2015) and Roth et al. (2020)). It is fair to assume, then, that people’s (implicit or explicit) political biases may also lead them to systematic errors in the labelling of CV data that are subsequently used for AI training purposes, which can result in algorithmic political bias.

(2) Unrepresentative sampling

Algorithmic bias, in general, can also emerge when the AI training data are labelled correctly but remain unrepresentative, yielding models that perform worse on under-sampled groups. For instance, the training data for an algorithm used for diagnosing COVID-19 symptoms might unintentionally be drawn from hospitals frequented predominantly by White, rich, male individuals. The algorithm is then trained ‘using unrepresentative or incomplete data from electronic health records that reflect disparities in healthcare access and quality’, resulting in an AI system that is likely to more frequently mislabel individuals not belonging to the dominant group and so will ‘reflect, repeat, and compound pre-existing structural discrimination’ (Leslie et al., 2021, p. 2). Similarly, if a hiring algorithm for AI job positions is trained on recruitment data only from Silicon Valley, since people on the radical left and conservatives tend to be underrepresented in this area (Broockman et al., 2019; Tiku, 2018), the algorithm may subsequently downgrade their AI job applications, as their political orientation has in the (unrepresentative) sample a negative statistical effect on the predicted probability of working in the field, resulting in algorithmic political biases.

(3) Mirroring existing social inequalities

Finally, algorithmic biases, in general, can also emerge even when training data are correctly labelled and representative. This is because social inequalities (often resulting from historical injustices) are common in many social environments (Lee et al., 2019). AI models that accurately represent these environments will also reflect these negative aspects and may in their processing replicate them (Noble, 2019). To see how this might lead to algorithmic political bias, in particular, consider an example.

Suppose that an algorithm for allocating university scholarships is trained with data from previous students’ CVs, capturing their demographics, grades, and political orientation (e.g. the CVs contain cues of liberal or conservative campaigning). The algorithm learns to map these data onto students’ subsequent achievements. Suppose further that while the data are representative and correctly labelled, they come from an environment where (e.g. due to structural disadvantages) both female and liberal students pattern with those who are low academic achievers. During the training, the algorithm thus forms a model that connects being female or liberal with lower achievement, and being male or conservative with higher achievement. Suppose that the algorithm is then given new CV data from two groups of students applying for scholarships, where individuals from both groups have identical grades (and gender) but those in one group are also liberal whereas the others are conservative. Using its predictive model, the algorithm systematically classifies the liberal students as less likely to be high achievers based on their political orientation and allocates the scholarship to students from the other group.

In this example, political orientation (and gender) reliably correlates with academic achievement. But since the algorithm bases its verdict concerning the scholarship on students’ political orientation, its output is clearly biased. After all, if we replaced ‘liberal’ in the example with ‘female’, the output would be treated as unfair and biased too. Since, in the case at hand, the algorithm deviates from a moral standard by unfairly^4^ privileging conservative over liberal students, it instantiates algorithmic political bias even though no human biases were involved in the AI training. In sum, algorithmic political bias can emerge in some of the same ways in which algorithmic gender and racial biases arise. And it can lead to some of the same ethically problematic outcomes in, for instance, job hiring or scholarship contexts.

## Distinctive Features of Algorithmic Political Bias

Having argued that algorithmic political biases share some key features with algorithmic gender and racial biases, there are also important differences. They can best be illustrated by first comparing the human equivalents of these biases.

### Human Gender and Racial Biases vs. Political Biases

In many cases, people’s political biases are likely to have a stronger impact on cognition and behaviour than, for instance, implicit racial biases when both political orientation and racial identity are known. For example, Iyengar and Westwood (2015) tested the strength of political bias (Democrat vs. Republican) compared to racial bias (European vs. African-American) by using an IAT measuring the reaction time people needed to associate Democrats vs. Republicans and Europeans vs. African-Americans with positive or negative attributes. They found that negative cross-political associations were significantly faster (hence more automatic) than negative associations related to African-Americans.

Iyengar and Westwood also asked people to decide on the basis of CVs that contained markers of either racial or political identity whether to award a scholarship to a student applicant. They found that for both Democratic and Republican participants political orientation had a more significant, negatively biasing impact on the decisions than racial identity. Relatedly, while there is some evidence that job-related information such as knowledge and skills can attenuate the effect of ethnicity and gender on hiring decisions, Roth et al. (2020) found that ‘political similarity processes continued to significantly influence hire-ability ratings even when information about applicant qualifications and accomplishments were included in the design and analyses’ (p. 482). What might explain these differences?

Iyengar et al. (2019) suggest that political biases are more pronounced because ‘[u]nlike race, gender, and other social divides where group-related attitudes and behaviors are subject to social norms […], there are [in the USA] no corresponding pressures to temper disapproval of political opponents. If anything, the rhetoric and actions of political leaders demonstrate that hostility directed at the opposition is acceptable and often appropriate’ (p. 133). For instance, US media outlets often present evidence of overt hostility among political opponents, including unrestrained exchanges of insults that are largely accepted, sometimes applauded (think of Trump’s ‘Crooked Hillary’,^5^ or De Niro’s ‘F*** Trump!’^6^ claims; Moody-Adams, 2019).

To be sure, the USA has a two-party structure and frequent political campaigning might produce a unique political in-group vs. out-group dynamic, potentially magnifying political hostility in ways less likely in political systems with multiple parties (Lelkes & Westwood, 2017). However, research suggests that while the extent of political polarization and hostility does differ internationally, it is both common (think of Brexiteers vs. Remainers, populists (anti-immigration) vs. cosmopolitans (pro-immigration advocates), etc., Druckman et al., 2020) and relatively stable across various types of democratic countries, including ones with many different political parties and viewpoints (Westwood et al., 2018). In fact, some studies found that ‘affective [political] polarization is [also] acutely present in European party systems, as partisans are often extremely hostile towards competing parties’ (Reiljan, 2020, p. 1).

It should not be surprising that some degree of aversion, hostility, and incivility between people of different political orientations is common and largely tolerated in Western democracies. This is because it can have positive consequences such as prompting political engagement of the electorate. It can also serve as a ‘tool of insurrection’, and calls for political civility might have the ‘negative function’ of ‘silencing or subjugating a marginalized group’ (Jamieson et al., 2017, p. 212). Kennedy (2001) goes further arguing that the ‘civility movement is deeply at odds with what an invigorated liberalism requires: intellectual clarity; an insistence upon grappling with the substance of controversies; and a willingness to fight loudly, openly, militantly, even rudely for policies and value’. Moreover, while gender and racial identity are (often) not chosen, people’s political orientation is commonly^7^ a matter of choice that might involve adopting value judgements that offend and harm others (e.g. judgements on whether one supports gay marriage, White supremacy, etc.; Roth et al., 2020). Given these points, some open and tolerated aversion amongst political opponents should be expected in a functioning democracy and might often be justified. This evidently (and rightly) does not hold for anyone’s aversion against people based on their gender or racial identity.

As understandable as this difference may be, it also leads to an important problem with political bias. The reason is that this difference makes it more likely that political bias and hostility can *spill over* from domains where they are acceptable into domains of judgement- and decision-making where they are widely viewed as unacceptable. Studies in which political biases affected decisions on scholarship award (Iyengar & Westwood, 2015), job hiring (Roth et al., 2020), or research manuscripts (Abramowitz et al., 1975; Ceci et al., 1985) illustrate this. In all these situations, basing one’s verdict on the political orientation of candidates or manuscripts rather than their competence or quality would clearly be viewed as unacceptable even by many people who see some political hostility in the political arena as acceptable part of democratic societies.

Similarly, many people may hold that, in academia, political values should be irrelevant and the focus should be on objectivity and competence (Haidt, 2016). Yet, surveys with US (Yancey, 2011; Shields & Dunn 2016) and international samples (Inbar & Lammers, 2012; Peters et al., 2020) found that many academics in the sciences and humanities openly expressed willingness to discriminate against colleagues with a political orientation opposite to their own. If such overt bias had been found against, for instance, women then perhaps shock waves would have gone (rightly) through academia. Yet, this hardly happened in the political orientation case. These points suggest that while there are social norms against gender and racial biases that strongly penalize people for such biases across domains, there are no equally powerful and wide reaching norms doing the same for political bias. Since political biases are largely left unchecked in many domains in the public sphere (e.g. the media, politics, campaigns), they can more easily and likely bleed into other domains where they are just as problematic as gender and racial biases (hiring, paper reviewing, etc.).

### Revisiting Algorithmic Political Bias

If human political biases have the features just mentioned, this has implications for the theorizing about, and the risks posed by, *algorithmic* political biases. Specifically, the preceding points provide reasons to believe that, in comparison to algorithmic gender and racial biases, algorithmic political biases are particularly likely to emerge and especially hard to detect and eradicate.

Notice first that AI system developers and managers are not generally apolitical. They often have (just as everyone else) certain political identities (Broockman et al., 2019). Since the existence of political biases in people in general is well documented (Iyengar et al., 2019; Jost et al., 2009), we should expect AI developers and managers, too, to have certain biases protecting or favouring their own political views. As noted above, these biases may then (just as their gender or racial biases) affect, for instance, their labelling of training data for algorithms that draw inferences about people or contents (e.g. for profiling job applicants, online recommendations, etc.). Indeed, since implicit political biases can affect people’s responding more strongly than, for instance, racial biases (Iyengar & Westwood, 2015), there is reason to believe that these biases will also more likely and more automatically lead individuals to link negative features with their political opponents when labelling relevant AI training data. This increases the chances that these biases become embedded in algorithms.

Additionally, when political biases influence the labelling and selection of AI training data, these effects are likely to be more difficult to detect for the people involved than if gender or racial biases did so. This is because there are strong and comprehensive social norms against the second kind of biases that make them salient and boost people’s attention to their potential effects. But there are no equally powerful and wide reaching norms to make political biases salient and motivate AI developers and managers to attend to them. This should reduce their ability to check for and recognize these biases during the labelling and selection of AI training data.

It might be suggested that while in politics, political aversion is often viewed as acceptable, when it comes to AI developers’ task of classifying individuals or contents for ML purposes, this is a different context. Discrimination based on features, such as political orientation, that are causally irrelevant for a target variable may not be tolerated by people working in the field of AI at all.

However, as noted, survey data indicate that in other domains, for instance, academia, where we would perhaps also strongly expect people to constrain their politically biased responding, this does not happen the way one would think either (Inbar & Lammers, 2012; Peters et al., 2020; Yancey, 2011). There is thus evidence that political aversion and readiness to discriminate against political opponents sometimes migrate from domains where they are acceptable into domains where people are expected to keep them in check and not discriminate others based on features that are causally irrelevant. The reliability of the intuition that the domain of AI development or management may be impervious to such spill-over effects becomes thus questionable.

Such effects are likely to be more common in countries in which political polarization and cross-political hostility are pervasive in politics and the media. This is because increased exposure to these social factors (just as increased exposure to media violence) may desensitize people and increase their tolerance threshold for them (Krahé et al., 2011) . Crucially, one of the countries currently dominating much of the most influential AI developments, namely the USA (Savage, 2020), fits the bill of a nation with strong, widely broadcast political polarization (Finkel et al., 2020; Talisse, 2019).

Moreover, even in cases when AI training data are correctly labelled and representative, compared to algorithmic gender and racial biases, algorithmic political biases are still particularly likely to result and produce harmful effects. The reason is that in an environment in which social norms strongly curb gender and racial biases, these kinds of biases are less likely to become part of predictive models that simply reflect this environment than in an environment in which no such norms exist. By extension, since Western societies currently contain no equally strong domain-general social norms against political biases, such biases should be more likely to become part of predictive AI models that just reflect these environments.

Also, since political aversion, hostility, and incivility are in many domains (campaigns, politics, the media) in Western societies to some extent tolerated, the dangers tied to algorithmic political biases in AI systems that are trained to operate in *other* domains (e.g. hiring) are particularly likely to be underestimated or downplayed. Underestimations may be especially common amongst people whose political opponents are targeted by these biases, as the power of the rationale that the ‘enemy of my enemy is my friend’ is well documented (Aronson & Cope, 1968). Additionally, a recent meta-analysis of studies on political bias found that, for instance, liberal and conservative study participants equally evaluated otherwise identical information more favourably when it supported their prior political identity vs. when it threatened it (Ditto et al., 2019). There may thus be much less societal unity on whether or how to tackle algorithmic political biases (compared to gender and racial biases), making it harder to appreciate and identify their potential dangers.

Relatedly, there is a clear consensus in democratic nations that all gender and racial discrimination should be eradicated (West et al., 2019). Indeed, discrimination based on gender or racial identity is *illegal* in Western societies (Chopin & Germaine, 2017). Yet, when it comes to political discrimination, this is less clear. Discrimination based on some political orientations (e.g. extremist views) is legal, as they violate fundamental rights of others. More generally, unlike in the EU,^8^ in some US states, ‘private employers may discriminate against their employees and job applicants based on political beliefs and some political activities’: ‘political behaviors and beliefs are not protected classes under the major employment anti-discrimination laws’ (Spiggle, 2021). Similarly, the UK government states: ‘It is not automatically unfair to dismiss someone because of their political beliefs or political groups they belong to’.^9^Since political discrimination is not ethically or legally problematic per se, efforts to track and determine how to deal with it and its algorithmic instantiations become more complex, making it more difficult to readily and clearly see the related risks. In fact, just drawing the line between political orientations that are fair targets of aversion and those that are not is often challenging. Political views are on a spectrum between extremes, where setting a particular point as a ‘red line’ can become arbitrary, hotly debated, or based on prior value judgements that change over time (Ekström et al., 2020). The elusiveness and changeability of a clear demarcation line can make recognizing and tackling algorithmic political biases again particularly difficult.

### Why Algorithmic Political Bias May Be Worse than Human Political Bias

Most of the concerns just outlined also apply to human political biases. But there is an important point that suggests that they are significantly more pressing when it comes to algorithmic political biases. The point harks back to the detection of political orientations.

It could be argued that someone’s political leaning, unlike their gender or racial identity, is much less detectable on, say, their CVs, in names, or their faces. The chances that a mislabelling of AI training data, unrepresentative sampling, or an AI’s mirroring of existing social inequalities may result in algorithms correlating political orientations with negative outcomes may thus be much lower than in the case of gender and racial identity. In fact, it seems that people can, if they want to, relatively easily conceal their political identity in everyday life and social environments, at the workplace, and so on (resulting in ‘invisible’ diversity) (Clair et al., 2005). They may just refrain from expressing their views (Shields & Dunn, 2016).

However, AI algorithms changed this. For content personalization purposes, various websites (Google, Facebook, etc.) now employ algorithms specifically trained to infer people’s attitudes, including political orientations (Hinds & Joinson, 2019), from their ‘digital footprints’ (e.g. their clicks, news browsing, etc.) even when website users do not explicitly express any political statements (Lambiotte & Kosinski, 2014; Vincent, 2016). For instance, some of these algorithms can infer people’s political orientations simply from a set of their Facebook ‘likes’ where these ‘likes’ are not themselves indicative of any particular political view (Youyou et al., 2015). Even the point that people’s political orientations cannot be read off from their faces is now questionable. A recent study published in *Science* (Kosinski, 2021) found that some existing facial recognition algorithms (used by e.g. the London Metropolitan Police; Santow, 2020) can be trained to ‘expose individuals’ political orientation, as faces of liberals and conservatives consistently differ’: ‘Political orientation was correctly classified in 72% of liberal–conservative face pairs, remarkably better than chance (50%), human accuracy (55%), or one afforded by a 100-item personality questionnaire (66%). Accuracy was similar across countries (the U.S., Canada, and the UK), environments (Facebook and dating websites), and when comparing faces across samples’ (Kosinski, 2021, p. 1). That is, single facial images, which are often easily accessible to the public on Facebook, LinkedIn, etc., can already ‘reveal more about a person’s political orientation than their responses to a fairly long personality questionnaire’ (ibid). Some algorithms can thus detect people’s political leanings even in cases when individuals do not want that to happen and would prefer to hide the relevant cues in human social interactions. As a result, the more people become subjected to these algorithms (e.g. when companies assess job applicants’ social media profile, use AI for face recognition, etc.), the smaller the space in which they can avoid becoming the targets of political biases by concealing their political orientation. This is because even though algorithms trained to detect political orientations of individuals, groups, or contents need not also be biased against them, they can provide *human* decision-making agents with ready insights into others’ otherwise often hidden political orientation. And if these human agents subsequently process this information in their decision-making including, for instance, in the labelling of AI training data, it may trigger their political biases, which in turn can influence their data labelling, and result in algorithms inheriting political biases in the ways outlined above.

In fact, since many algorithms already routinely track people’s political orientations (for website personalization purposes), and data sharing between website algorithms is common (Rodriguez, 2020), it is only to be expected that future (potentially even already some existing) job-recruitment algorithms will also draw on digital footprint data to inform hiring decisions and use people’s political orientations as predictors. If such an algorithm has initially learned, for instance, through CV cues (from people’s face pictures, past campaigning, etc.) that in a particular company, individuals with a certain political orientation are not hired, then even equally qualified applicants who intentionally omit any marker of this orientation in their submitted applications might still be treated worse by the algorithm when it detects signs of that orientation in their digital footprint data (faces, etc.). While (AI-unaided) human political biases would in these situations be undercut, *algorithmic* political biases can still operate. The potential harm related to them in the future is hence likely to be significantly higher than that connected to human political biases.

### Clarifications

In considering the preceding argument, two points should be noted. First, there are international differences with respect to norms against political biases, discrimination, and polarizations (Boxell et al., 2020; Westwood et al., 2018). In some democratic countries, the normative constraints on them might be stronger than in others (Finkel et al., 2020). For instance, while in the UK^10^ and many states of the USA (Spiggle, 2021), political belief is not a protected characteristic (like gender or racial identity), in Germany^11^ and the EU,^12^ it is. Correspondingly, in some countries, AI system developers and managers may be more sensitive to their own and their algorithms’ potential political biases than in other countries. This does not undermine the importance of the argument here because the general difference in weaker social restrictions on people’s aversion against political opponents (vs. their aversion against individuals with a different gender or racial identity) is present and robust across democratic nations. Moreover, as noted, the country that is currently leading the world in AI developments, the USA (Savage, 2020), is also one of the democratic societies currently leading the world in people’s political division, polarization, and limited social checks on them (Boxell et al., 2020; Iyengar et al., 2019). In the USA, ‘political tribalism’ (i.e. people’s viewing themselves as belonging to either the group of liberals or the group of conservatives and displaying overt aversion against political out-group members) is at an all-time high and pervasive (Finkel et al., 2020). The argument here should thus be especially relevant for the AI community in the United States.

Second, it might be objected that the argument overgeneralizes because, in fact, *any* predictor that should be normatively irrelevant for an individual’s classification or evaluation could become important in an ML model. It could be hair colour, baldness, wearing glasses, consumption habits, etc.—political orientation is only one. Yet, there are also, for instance, no legal protections against discrimination by any of these characteristics either. Moreover, probing whether any one of them ends up being associated with the target variable could be equally hard, as one would also need to inspect the model and possibly the AI training data to uncover this bias and its source. It may therefore seem unjustified to claim that specifically bias based on political orientation is harder to detect.

However, there are significant differences. While hair colour (Stollznow, 2021), baldness (Kranz et al., 2019), or wearing glasses can be targets of human and algorithmic biases (Seo et al., 2021), such biases are likely much less pronounced and established (Walline et al., 2008) than gender, racial, and political biases. People are hardly ever denied jobs, loans, and flat leases, or stopped and searched because they are blonde, or wear glasses (Fogg, 2013). Relatedly, there is no open hostility against, say, ginger-haired, or bald people in, for instance, academia. And while political orientations come with value systems that often define people’s identity and determine whether we trust and cooperate with them (Koch et al., 2016), this is not the case for hair colour, etc. Correspondingly, there are deep and persistent social divides between political opponents in many countries, fuelling political biases, but similar divides between (e.g.) blondes/non-blondes are absent. Moreover, while political hostility and discrimination is in some domains tolerated, there is arguably no domain (in democracies) in which hostility or discrimination against, say, ginger-haired or glass-wearing people is tolerated, which significantly decreases the chances of strong correlations between such features and negative outcomes.^13^ This should make the relevant biases much less likely to be passed on from humans (e.g. through mislabelling of AI training data) to machines.

## Distinctive Challenges for Mitigating Algorithmic Political Bias 

The literature contains many concrete recommendations on how to reduce algorithmic gender and racial biases, including interventions that use debiasing algorithms (for data pre-processing before training, in-processing during training, or post-processing after training; Amini et al., 2019; Bellamy et al., 2019). But some common, more general suggestions might face particular challenges when it comes to algorithmic political bias.

For instance, one basic strategy that many researchers have proposed against algorithmic gender and racial biases is to diversify the field of AI (including developers, managers, researchers) regarding people’s gender and racial identity (Hagerty & Rubinov, 2019). The more heterogeneous the teams developing AI algorithms and researching their implications, the higher the likelihood that biases in data selection, labelling, and programming are detected and counteracted (Cowgill et al., 2020). Interacting with diverse colleagues can also reduce individuals’ own biases that may affect their interactions with algorithms (Bodenhausen et al., 2009). Similarly, the more *politically* diverse groups of AI developers, managers, and researchers are, the higher the likelihood that algorithmic political biases are kept in check too.

However, implementing political diversity in teams working on AI can be particularly difficult. This is because people with certain political identities may not want to enter AI research because of their convictions. For instance, AI entrepreneurs and programmers in AI hot spots such as Silicon Valley often oppose government interventions in markets, government support for labour unions, or worker and consumer protections (Broockman et al., 2019). This can deter people from the radical left to consider working in AI development because their political conviction jars with such opposition. Relatedly, since Silicon Valley is predominantly politically (moderately) liberal with most AI and Internet companies endorsing progressive viewpoints (ibid), conservatives might feel ‘out of place’ there (Tiku, 2018). Additionally, if political polarization is widespread and persistent within society itself, political diversity measures in AI teams may result in social conflicts undermining the cooperation of team members (Eagly, 2016).

Another approach to reducing algorithmic political biases might be to change the wider social context and social norms that govern people’s responses towards their political opponents and partly account for the stronger impact of political biases on cognition and behaviour. Making political orientation a protected characteristic (in places where this is not already the case) might be an option, and normative frameworks may be developed and promoted that encourage civility in political debates and interactions with political opponents. With stronger social norms to curb political hostility at workplaces, in the media, politics, and so on, people, including AI developers and managers, may become more motivated to monitor their own responding when encountering political opponents or contents that contradict their own political values. This can reduce the chances that political biases become passed on to algorithms.

However, as noted, political incivility, aversion, and hostility can be positive for a functioning democracy, providing ‘tools of insurrection’ (Jamieson et al., 2017, p. 212), and may even be required for an ‘invigorated liberalism’ (Kennedy, 2001). Indeed, instituting social norms for more political toleration may also in some cases inadvertently result in silencing stigmatized minorities (Jamieson et al., 2017). Changes to the existing norms that govern people’s interactions with political opponents or their opinions so as to reduce political bias, in general, and algorithmic political bias, in particular, should therefore be carefully assessed. More interdisciplinary research is needed to analyse how the relevant existing social norms can be changed without negatively affecting processes that belong to a healthy democracy.

## Conclusion

Algorithmic bias may target different dimensions of social identity with potentially many different ethically and epistemically important implications depending on which one is targeted. Algorithmic bias against political orientations has remained largely unexplored in the AI literature. While various phenomena might be interpreted as algorithmic political bias, this paper focused on cases in which algorithms make predictions and decisions based on people’s political orientation in contexts where this feature should be irrelevant. I argued that AI algorithms can become biased in this way against the political orientations of people (and contents) in some of the same ways in which they can acquire gender or racial biases. And they may subsequently become used in, for example, job-recruitment contexts, where they can produce some of the same harm (e.g. unfair decisions). However, despite these commonalities, while there are powerful, domain-general social norms against gender or racial biases, this is not equally the case for political biases. These biases can thus more strongly influence people’s cognition and behaviour, which increases the likelihood that they become transferred to algorithms through, for instance, the mislabelling of AI training data, unrepresentative sampling, or simply an AI system’s mirroring of social reality. The difference in social norms highlighted here may also make it more difficult for people to detect and counteract algorithmic political biases because it makes these biases less salient as problematic phenomena. Worse still, while people could previously avoid becoming the target of these biases by concealing their political orientation, some algorithms now allow uncovering people’s political viewpoints against their will, making individuals more vulnerable to the related biases than before. Changes to the social norms that govern people’s responses to political opponents may help mitigate algorithmic political biases. But these norms also have desirable aspects. This significantly complicates the task of tackling algorithmic political bias in ways that should be taken into account by AI developers, managers, and ethicists.

## Data Availability

Not applicable.

## Footnotes

1 As indicated with the qualifier ‘no equally powerful norms’ here, I do not deny that there are also (potentially strong) social norms against political bias, hostility, etc. The claim is that these norms are not *equally powerful* for reasons outlined in Section 4. I am grateful to an anonymous reviewer for prompting me to be clearer on this.

2 There are many different kinds of AI classification errors (e.g. false positives, false negatives, generalization errors) and they might arise in different ways (Meek, 2016; Mohri et al., 2018).

3 ML may involve many different programs; e.g. supervised, unsupervised, or reinforcement learning; see Burrell (2016) for details.

4 It might not be intrinsically unfair to use features causally irrelevant for academic achievement in predictions of academic achievements and scholarship allocation. What matters here is just that it is intuitively plausible that we would view the example as a case of unfair treatment.

5 https://www.theguardian.com/us-news/shortcuts/2018/apr/17/presidents-nicknames-slimeball-comey-former-fbi-director

6 https://www.theguardian.com/film/2018/jun/11/robert-de-niro-wins-ovation-fuck-trump-speech-tony-awards

7 But this may not always (fully) be the case (see, e.g. Funk et al., 2013; Kalmoe & Johnson, 2021).

8 Article 21 of the EU Charter of Fundamental Rights says: ‘Any discrimination based on any ground such as sex, race, colour, ethnic or social origin, genetic features, language, religion or belief, political or any other opinion, membership of a national minority, property, birth, disability, age or sexual orientation shall be prohibited’. See https://fra.europa.eu/en/eu-charter/article/21-non-discrimination

9 https://www.gov.uk/dismiss-staff/unfair-dismissals

10 https://www.gov.uk/dismiss-staff/unfair-dismissals

11 See https://www.antidiskriminierungsstelle.de/SharedDocs/downloads/EN/publikationen/agg_wegweiser_engl_guide_to_the_general_equal_treatment_act.pdf?__blob=publicationFile

12 See https://fra.europa.eu/en/eu-charter/article/21-non-discrimination

13 Nonetheless, the main problem highlighted here, i.e. that of unjustifiable predictors and proxies in the ML classifier models, is of a more general nature and not limited to political orientation. I will leave the project of relating algorithmic political bias to the broader literature on this issue (e.g. Johndrow & Lum, 2019; Mehrabi et al., 2020) for another occasion.
